# Assessment of the Mechanical and Thermal Properties of Injection-Molded Poly(3-hydroxybutyrate-*co*-3-hydroxyhexanoate)/Hydroxyapatite Nanoparticles Parts for Use in Bone Tissue Engineering

**DOI:** 10.3390/polym12061389

**Published:** 2020-06-21

**Authors:** Juan Ivorra-Martinez, Luis Quiles-Carrillo, Teodomiro Boronat, Sergio Torres-Giner, José A. Covas

**Affiliations:** 1 Technological Institute of Materials (ITM), Universitat Politècnica de València (UPV), Plaza Ferrándiz y Carbonell 1, 03801 Alcoy, Spain; juaivmar@doctor.upv.es (J.I.-M.); luiquic1@epsa.upv.es (L.Q.-C.); tboronat@dimm.upv.es (T.B.); 2 Novel Materials and Nanotechnology Group, Institute of Agrochemistry and Food Technology (IATA), Spanish National Research Council (CSIC), Calle Catedrático Agustín Escardino Benlloch 7, 46980 Paterna, Spain; 3 Institute for Polymers and Composites, University of Minho, 4804-533 Guimarães, Portugal

**Keywords:** P(3HB-*co*-3HHx), nHA, nanocomposites, mechanical properties, bone reconstruction

## Abstract

In the present study, poly(3-hydroxybutyrate-*co*-3-hydroxyhexanoate) [P(3HB-*co*-3HHx)] was reinforced with hydroxyapatite nanoparticles (nHA) to produce novel nanocomposites for potential uses in bone reconstruction. Contents of nHA in the 2.5–20 wt % range were incorporated into P(3HB-*co*-3HHx) by melt compounding and the resulting pellets were shaped into parts by injection molding. The addition of nHA improved the mechanical strength and the thermomechanical resistance of the microbial copolyester parts. In particular, the addition of 20 wt % of nHA increased the tensile (E_t_) and flexural (E_f_) moduli by approximately 64% and 61%, respectively. At the highest contents, however, the nanoparticles tended to agglomerate, and the ductility, toughness, and thermal stability of the parts also declined. The P(3HB-*co*-3HHx) parts filled with nHA contents of up to 10 wt % matched more closely the mechanical properties of the native bone in terms of strength and ductility when compared with metal alloys and other biopolymers used in bone tissue engineering. This fact, in combination with their biocompatibility, enables the development of nanocomposite parts to be applied as low-stress implantable devices that can promote bone reconstruction and be reabsorbed into the human body.

## 1. Introduction

Bone fracture is one of the most common injuries. Bone regeneration encompasses three stages, namely inflammation, bone production, and bone remodeling [1]. During the latter, it is extremely important to expose the bone to the natural load-bearing conditions associated to its function [2]. Currently, titanium alloys such as Ti-6Al-4V are the most used for the manufacture of orthopedic fixing devices and bone implants due to their excellent biocompatibility and high mechanical resistance [3]. However, they prevent the bone from being subjected to the required mechanical loadings [4]. Indeed, while natural bone has a modulus ranging between 8 to 25 GPa, metals have a modulus of 110–210 GPa, which results in the load being imparted onto the device rather than the bone which then causes a localized decrease in bone mineral density [5]. Meanwhile, metal ion leaching increases inflammation and irritation around the implant [6]. As a result, there is often a need for a second surgery to remove the fixation device, leading to higher medical costs and greatly increased patient discomfort. A current alternative is the use of fixation devices that metabolize in the human body after fulfilling their function [7]. In particular, the use of biopolymers with biocompatibility and reabsorption capacities is very promising [8]. Biocompatibility involves the capability of a given substance to perform with a suitable host response in a particular use. Furthermore, no substance or material can be “biocompatible” if it releases cytotoxic substances. The degradation process of a given biopolymer within the human body consists of two phases. First, the biopolymer chains break, either as a consequence of hydrolysis or due to the action of a body enzyme. Thereafter, the human body assimilates the fragments. For this purpose, either a phagocytosic or metabolic process develops [9]. Surface porosity, shape, and tissue environment, including chemical build-up of the materials, play a significant role in biocompatibility [10,11].

For the past few decades, polymers of the polyhydroxyalkanoates (PHAs) family have been paving the way for the development of new biomedical products. These microbial biopolyesters degrade when exposed to marine sediment, soil or compost. A vast number of microorganisms secrete extracellular PHA-hydrolyzing enzymes, so-called PHA depolymerases, to degrade PHA into their oligomers and monomers, which subsequently act as nutrients inside the cells [12]. Their potential as alternatives for the manufacture of a wide range of medical devices, such as absorbable sutures, surgical pins or staples, is well recognized on account of their biodegradable nature as well as disintegration by surface erosion [13]. Broadly, the biocompatibility of PHA materials can be differentiated into two categories, immunocompatibility and nonallergic response. The former involves the extent of antigenic resemblance between the tissues of various individuals that determines the acceptance or rejection of allografts. PHAs are essentially immunocompatible for use in medical applications, that is, their materials should not elicit harsh immune responses upon introduction into the soft tissues or blood of a host organism [14]. Indeed, 3-hydroxybutyrate (3HB), the main monomeric constituent of most PHAs, is a result of cellular metabolism that is formed by oxidation of fatty acid within the liver cells and it is a usual component of human blood [15]. Other previous studies have also revealed that PHA did not elicit an allergic response or any hypersensitive immune reaction [10,16].

Depending on the number of carbon atoms in the monomers, PHAs can be classified as short-chain-length PHAs (*scl*-PHAs; 3−5 C-atoms) and medium-chain-length PHAs (*mcl*-PHAs; 6−14 C-atoms). Generally, *scl*-PHAs are rigid and brittle, while *mcl*-PHAs have higher flexibility and toughness [17]. Poly(3-hydroxybutyrate) (PHB) is the simplest and most common member of the PHA family. However, the high brittleness of PHB and other *scl*-PHAs, such as poly(3-hydroxybutyrate-*co*-3-hydroxyvalerate) (PHBV) with less than 15 mol % fraction of 3-hydroxyvalerate (3HV), restricts their application in bone fixing devices [18]. In this regard, poly(3-hydroxybutyrate-*co*-3hydroxyhexanoate) [P(3HB-*co*-3HHx)], also referred as PHBH, represents a recent addition to the group of PHAs for biomedical applications. The introduction of the *mcl* 3-hydroxyhexanoate (3HHx) co-monomer into the polymer backbone of PHB significantly increases the flexibility and reduces stiffness [19]. Therefore, the macroscopic properties of P(3HB-*co*-3HHx) vary with the proportion of each monomer in the copolyester [20], in which the higher the 3HHx content, the higher the ductility [21]. Apart from the changes in the mechanical properties, the most remarkable transformation that P(3HB-*co*-3HHx) brings along is its ability to undergo enzymatic degradation by lipase [22], which is not seen in either PHB or PHBV. Prior experiments have shown that materials based on P(3HB-*co*-3HHx) and other *mcl*-PHAs have good biocompatibility for chondrocytes [23], nerve cells [24] as well as osteoblast and fibroblast cells [25,26]. This property should make P(3HB-*co*-3HHx) a suitable choice for several tissue engineering applications since it adds a further variable that can be used to tailor its degradation [27].

While PHAs are biocompatible substrates for cell propagation and are potentially an effective template for the repair of osseous and chondral defects, there is still a need to improve the mechanical strength, thermal resistance, and biological response of these biomaterials in order to make them more suitable for bone tissue engineering. Osteoconductive fillers can be introduced into polymer matrices with the aim of improving the mechanical properties and also accelerating the bone repair process by favoring the growth of bone cells inside the pores [28,29]. For example, calcium orthophosphates (CaPO_4_) have bioactive properties that increase bone cell proliferation, the so-called osteoinduction [30]. As a rule, both the mechanical resistance and bioactivity of composites prepared with collagen, chitin and/or gelatin, increase with increasing CaPO_4_ content [31]. Hydroxyapatite, Ca_5_(PO_4_)_3_OHCa_5_(PO_4_)_3_OH, which is the principal crystalline constituent of bone, shows a high degree of biocompatibility and good osteoconductive and osteoinductive properties. Therefore, hydroxyapatite nanoparticle or nanohydroxyapatite (nHA) is the most widely used “bioceramic” for the manufacture of medical devices and dental implants [32]. This fact is exemplified by the production of prostheses for cranial reconstruction using poly(methyl methacrylate) (PMMA)/nHA composites [33]. Indeed, nHA exists in the human bone in the form of nanometer-sized threads, thus ensuring biocompatibility. At present, it is mostly used to produce surface coatings, as its biomimetic mineralization enables the production of biomaterials with biomimetic compositions and hierarchical micro/nanostructures that closely mimic the extracellular matrix of native bone tissue [34,35].

Due to the well-known high bioactive properties in terms of bone regeneration of PHA- and nHA-based composites, this study aims to determine the physical properties of injection-molded parts made of P(3HB-*co*-3HHx)/nHA composites, for potential use as bone resorbable devices. To this end, different contents of nHA were incorporated into P(3HB-*co*-3HHx) and the mechanical, thermal, and thermomechanical properties were analyzed and compared to some metal alloy-based solutions currently available in the biomedical field. As a first, the parts showed sufficient dimensional and thermal stability for bone tissue engineering and their elasticity was nearer to that of the natural bone when compared to the metal alloys used for bone implants.

## 2. Materials and Methods

### 2.1. Materials

P(3HB-*co*-3HHx) copolymer was supplied by Ercros S.A. (Barcelona, Spain) as ErcrosBio PH110. The ratio of 3HHx in the copolyester is ~10 mol % and its number average molecular weight (M_n_) is 1.22 × 10^5^ g/mol. It shows a melt flow index (MFI) of 1 g/10 min (2.16kg/160 °C) according to the ISO 1133-2 standard and a true density of 1.20 g/cm^3^ following the UNE EN ISO 1183-1 standard. Hydroxyapatite synthetic nanopowder was procured from Sigma-Aldrich S.A. (Madrid, Spain) with commercial reference 677418. According to the manufacturer, it presents the following properties: particle size < 200 nm, surface area > 9.4 m^2^/g by Brunauer-Emmett-Teller (BET) analysis, purity ≥ 97%, and molecular weight (M_W_) of 502.31 g/mol.

### 2.2. Preparation and Processing of P(3HB-co-3HHx)/nHA Parts

Both P(3HB-*co*-3HHx) pellets and nHA powder were dried separately for at least 6 h at 80 °C in a dehumidifying oven from Industrial Marsé S.A. (Barcelona, Spain). The materials were then pre-mixed manually in closed zip-bags at the ratios presented in Table 1.

The different P(3HB-*co*-3HHx) and nHA mixtures weighing 800 g were melt-compounded using a co-rotating twin-screw extruder from Dupra S.L. (Castalla, Spain). It features two screws with a diameter (D) of 25 mm and a length-to-diameter ratio (L/D) of 24, while the modular barrel is equipped with 4 individual heating zones coupled to a strand die. Further details of the extruder can be found elsewhere [36]. Extrusion was performed with a screw speed of 20–25 rpm to prevent material degradation due to shear-induced viscous dissipation, a feed of 1.2 kg/h, and a barrel set temperature profile of 110–120–130–140 °C from hopper to die. The extruded filaments were cooled down in an air stream and pelletized using an air-knife unit. 

Test parts for characterization were obtained by injection molding. The equipment (Meteor 270/75, Mateu & Solé, Barcelona, Spain) was operated with a barrel set temperature profile of 115–120–125–130 °C from hopper to nozzle, with the mold kept at 60 °C. An injection time of 1 s was used to avoid material degradation by shear-induced viscous dissipation. The clamping force was 75 tons and the cooling time was set at 60 s. Parts with a thickness of approximately 4 mm were obtained for characterization. Since P(3HB-*co*-3HHx) develops secondary crystallization with time, the parts were allowed to age for 14 days at room temperature prior to characterization.

### 2.3. Mechanical Tests

Uniaxial tensile tests were performed according to the ISO 527-2: 2012 standard using a universal testing machine ELIB-50 (Ibertest S.A., Madrid, Spain) fitted with a load cell of 5 kN and using a 3542-050M-050-ST extensometer from Epsilon Technology Corporation (Jackson, WY, USA). Flexural properties were determined following the ISO 178: 2011 standard using the same equipment. Both tests were carried out at 5 mm/min using 150 mm × 10 mm × 4 mm parts. Charpy impact tests were performed following the ISO 179-1: 2010 standard. Samples with a V-shaped notch with a radius of 0.25 mm and dimensions 80 mm × 10 mm × 4 mm were subjected to the impact of a 1-J pendulum impact tester from Metrotec S.A. (San Sebastián, Spain). Shore hardness was measured with a 673-D durometer (J. Bot Instruments, Barcelona, Spain), following the ISO 868: 2003 standard. At least six parts were tested for each mechanical test.

### 2.4. Thermal Tests

Samples weighing 5–10 mg were analyzed by differential scanning calorimetry (DSC) in a Q200 from TA Instruments (New Castle, DE, USA) to study the thermal transitions. The samples were subjected to a three-stage thermal cycle in which the samples were first heated from −50 to 200 °C and cooled down to −50 °C in order to eliminate the thermal history and then reheated to 200 °C. All the heating and cooling scans were performed at 10 °C/min. Testing was performed under inert atmosphere using a nitrogen flow of 50 mL/min. The degree of crystallinity (*X_C_max_*) was calculated using Equation (1) [37]:

(1) $$Xc_{max}=\left[\frac{\Delta H_{m}}{\Delta H_{m}\cdot \left(1-w\right)}\right]\cdot 100\%$$

where ∆*H*_m_ (J/g) corresponds to the melting enthalpy of P(3HB-*co*-3HHx), ∆*H*_m_^0^ (J/g) is the theoretical value of a fully crystalline of P(3HB-*co*-3HHx), taken as 146 J/g [38], an 1 − *w* indicates the weight fraction of P(3HB-*co*-3HHx) in the sample.

Thermogravimetric analysis (TGA) was performed to determine the thermal stability of the injection-molded parts. Samples weighing 10–20 mg were heated from 30 to 700 °C at a heating rate of 20 °C/min in a TGA 100 from Linseis Messgeräte GmbH (Selb, Germany) under nitrogen atmosphere with a flow rate of 25 mL/min. All thermal tests were carried out in triplicate.

### 2.5. Thermomechanical Tests

Injection-molded parts sizing 10 mm × 5 mm × 4 mm were subjected to a temperature sweep from −70 to 100 °C at a heating rate of 2 °C/min using a DMA-1 from Mettler-Toledo S.A. (Barcelona, Spain). Dynamic thermomechanical analysis (DMTA) was carried out in bending mode with a maximum bending strain of 10 µm at a frequency of 1 Hz and a force of 0.02 N. 

The dimensional stability of the parts was studied by thermomechanical analysis (TMA) in a Q400 thermomechanical analyzer from TA Instruments (New Castle, DE, USA). The applied force was set to 0.02 N and the temperature program was scheduled from −70 to 70 °C in air atmosphere (50 mL/min) at a constant heating rate of 2 °C/min. All thermomechanical tests were performed in triplicate.

### 2.6. Microscopy

The fracture surfaces of the injection-molded parts after the Charpy impact tests were analyzed by field-emission scanning electron microscopy (FESEM) (Oxford Instruments, Abingdon, UK) with an electron acceleration voltage of 2 kV. A gold-palladium coating was applied through sputtering (SC7620, from Quorum Technologies Ltd, East Sussex, UK). Additionally, to visualize the dispersion of nHA in the P(3HB-*co*-3HHx) matrix, the fracture surfaces were attacked with 6M hydrochloric acid (HCl) (37% purity, Panreac AppliChem, Barcelona, Spain) for 12 h to selectively remove nHA prior to observation [39].

### 2.7. Statistical Analysis

Statistical evaluation of the mechanical, thermal, and thermomechanical properties of P(3HB-*co*-3HHx)/nHA parts was carried out with the open source R software (http://www.r-project.org) with a Shapiro–Wilk test regarding a normal distribution for n < 1000. Tukey tests were performed to determine significant differences between the data on normally distributed data. In order to establish the non-parametric relationship between mechanical properties and nHA content in the parts, the Spearman’s correlation test was followed. The number of tested samples for each test is included in Table 2 and the level of significance was established as *p* < 0.05 in all cases.

## 3. Results and Discussion

### 3.1. Mechanical Characterization of the P(3HB-co-3HHx)/nHA Parts

**Figure 1:**
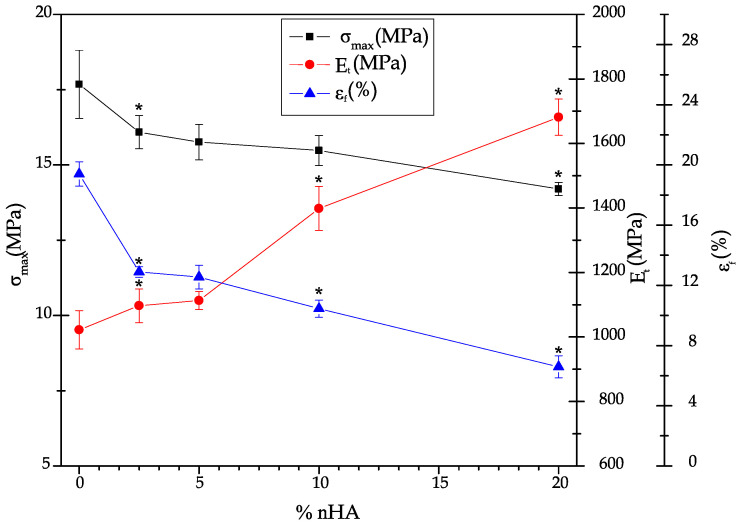
Evolution of the maximum tensile stress (σ_max_), tensile modulus (E_t_), elongation at break (ε_b_) in the injection-molded parts of poly(3-hydroxybutyrate-*co*-3-hydroxyhexanoate) [P(3HB-*co*-3HHx)] with the content of hydroxyapatite nanoparticles (nHAs). * Indicates a significant difference compared with the previous sample (*p* < 0.05)

**Figure 2:**
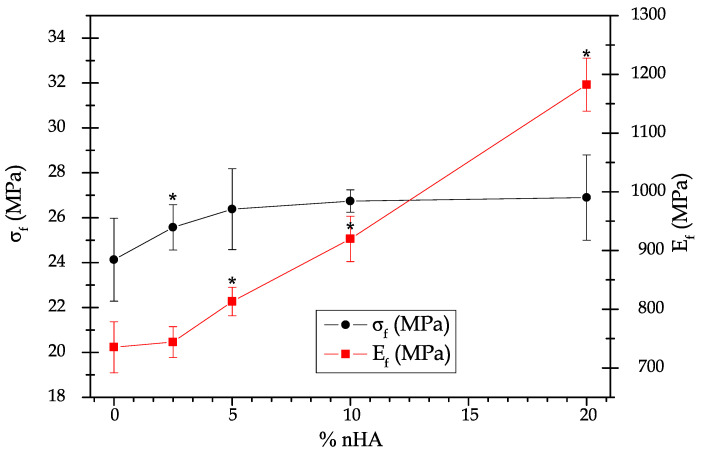
Evolution of the maximum flexural stress (σ_f_) and flexural modulus (E_f_) in the injection-molded parts of poly(3-hydroxybutyrate-*co*-3-hydroxyhexanoate) [P(3HB-*co*-3HHx)] with the content of hydroxyapatite nanoparticles (nHAs). * Indicates a significant difference compared with the previous sample (*p* < 0.05).

The data collected for the mechanical properties from the tensile, flexural, hardness, and impact Charpy tests of the neat P(3HB-*co*-3HHx) and P(3HB-*co*-3HHx)/nHA composite parts produced with the different compositions is summarized in Table 3. Figure 1 and Figure 2 display the effect of nHA incorporation on the tensile and flexural properties, respectively, whereas Table 4 shows the correlation coefficient (*r_s_*) and *p* for each mechanical property according to the Spearman’s test.

The tensile properties of the injection-molded P(3HB-*co*-3HHx) parts were relatively similar to those reported by Giubilini et al. [40], although the here-prepared materials were slightly less mechanically resistant and more ductile. These differences could be related to the 3HHx monomer content in the copolyester as well as to differences in processing. One can observe in both Table 3 and Figure 1 that the values of σ_max_ and ε_b_ decreased, while those of E_t_ increased with increasing nHA concentration in the nanocomposite parts. The Spearman’s test confirmed the existence of a trend between the tensile properties of the nanocomposites and the nHA content, showing a negative *r_s_* trend (inversely proportional correlation) for σ_max_ and ε_b_ and a positive trend (directly proportional correlation) for E_t_, while in all cases *p* < 0.05. In particular, the addition of 20 wt % of nHA produced a slight decrease of σ_max_ from 17.7 to 14.4 MPa, but an increase of nearly 64% in E_t_ (from approximately 1 to 1.7 GPa) accompanied with a significant loss of ductility (ε_b_ was reduced from 19.4 to 6.5%). The reduction in stress was probably caused by the poor interface adhesion between biopolymer and nanofiller. Higher interfacial adhesion can probably be promoted through the pretreatment of nHA with silanes [41], but it could negatively affect the biocompatibility of the parts. The increase in E_t_ was anticipated, since nHA forms highly rigid structures. Furthermore, as it will be discussed during the thermal characterization, the addition of nHA could promote higher degrees of crystallinity and, hence, higher stiffness. Although similar results have been reported earlier [42,43], the here-prepared parts showed higher ductility due to the use of a more flexible PHA. The decrease observed in stiffness with increasing nHA content can be attributed to insufficient wetting and impregnation of the nanoparticles by the polymer matrix, mainly due to particle agglomeration during manufacture or processing of the materials [44]. However, melt-mixing methodologies using co-rotating twin-screw extruders, as adopted here, can generally yield well-dispersed nanocomposites [45]. Ductility loss was expected since the presence of nHA can prompt polymer crystallinity, hindering chain mobility due to adsorption of biopolymer chains on the surface of the nanoparticles [46,47].

In Figure 2, it can be seen that the addition of nHA to P(3HB-*co*-3HHx) increased both σ_f_ and E_f_, particularly the latter. The former increased up to a content of 5 wt % of nHA and then became insensitive to higher nanoparticle contents, since the values showed no significant differences. Indeed, the Spearman’s test showed a positive correlation (*r_s_* > 0) for both E_f_ and σ_f_, however, for the latter, the statistical hypothesis should be rejected as p was higher than 0.05. Contrarily, the addition of 20 wt % of nHA caused an increase of approximately 60% of E_f_, as similarly observed above for E_t_. The resultant increase in mechanical strength can be related to the intrinsic high values of compressive strength and modulus of nHA, which are in the ranges of 500–1000 MPa and 80–110 GPa, respectively [48,49].

In comparison with the mechanical values of other degradable and non-degradable materials, the P(3HB-*co*-3HHx)/nHA parts produced in this study showed intermediate values to most biodegradable polymers and metal alloys. For instance, the E_t_ values of poly(ε-caprolactone) (PCL) and PLA materials range between 400–600 MPa [50] and 2−3 GPa [51,52], respectively, while other biodegradable copolyesters such as poly(butylene adipate-*co*-terephthalate) (PBAT) show significantly lower values [53]. However, PLA is a brittle polymer, which can limit its application in bone fixation devices, or any other biomedical device that would be subjected to local flexural stress or impacts. The values attained are relatively similar to those of poly(lactic-*co*-glycolic acid) (PLGA), that is, 1.4−2.8 GPa [54]. Indeed, PLGA is widely used in biomedical and pharmaceutical applications, but it shows longer degradation times, which can extend up to 12 months [55]. Regarding metal alloys, the E_t_ values of the most widely used stainless steels for implant fixing devices and screws, that is, SUS316L stainless steel and cobalt-chrome (Co-Cr) alloys, are around 180 GPa and 210 GPa, respectively [56]. Lower values have been reported for titanium (Ti) and its light alloys, such as Ti-6Al-4V ELI, which are also widely used for making implant devices, having a value of around 110 GPa [57]. As shown above, in comparison to metal alloys, the elasticity of the P(3HB-*co*-3HHx)/nHA composites prepared in this study is nearer to that of the natural bone, which is in the 8–25 GPa range [5]. Thus, from a mechanical point of view, their use in bone scaffolds and resorbable plates or screws looks promising.

As expected, hardness increased with the presence of nHA that, due to its ceramic nature, is highly rigid. The increase was significant at nHA contents higher than 2.5 wt % and this effect was statistically corroborated by Spearman’s test, showing a positive trend with an *r_s_* value of ~0.98. In addition, molecular mobility could be reduced due to the presence of the nanoparticles [58]. In particular, the incorporation of 20 wt % of nHA yielded an increase of 8% in hardness. A similar increase in Shore D hardness was reported by Ferri et al. [39] for PLA after the incorporation of nHA. In particular, it increased from 73.9, for neat PLA, up to 78.4, for the PLA composite containing 30 wt % of nHA. As also anticipated, the impact strength of the nanocomposites diminished significantly with increasing nHA content with significant differences between the samples, which was confirmed by the negative correlation obtained by the Spearman’s test (*r_s_* ≃ −0.84). For instance, the nanocomposite parts containing 20 wt % of nHA revealed an impact strength approximately three times lower than that of the neat P(3HB-*co*-3HHx) part, that is, it reduced from 5.1 to 1.7 kJ/m^2^. Lower values of impact strength were reported for V-notched injection-molded pieces of PLA, that is, 2.1 kJ/m^2^ [51]. In addition, significantly higher values have been described for Ti-6Al-4V, with a Rockwell hardness C (HRC) of 38 and approximately 112 kJ/m^2^ impact strength [59]. In the case of natural bone, toughness varies widely with age and type. For instance, the impact strength of the femora ranges from 4 to 70 kJ/m^2^ [60]. Therefore, the various mechanicals tests revealed a clear tendency towards a decrease in ductility and an increase in stiffness of the injection-molded parts with increasing nHA content, which are closer to those of the natural bone.

In summary, the here-developed P(3HB-*co*-3HHx)/nHA parts showed an improvement of the stiffness determined in terms of E_t_ and E_f_, in which a positive trend was observed in both cases (*r_s_* > 0). The ductile properties, that is, ε_b_ and impact strength, showed negative trends (*r_s_* < 0), which was ascribed to a chain mobility reduction that also contributed to a hardness increase of the nanocomposite, showing a positive trend in the Spearman’s test.

### 3.2. Thermal Characterization of the P(3HB-co-3HHx)/nHA Parts

**Figure 3:**
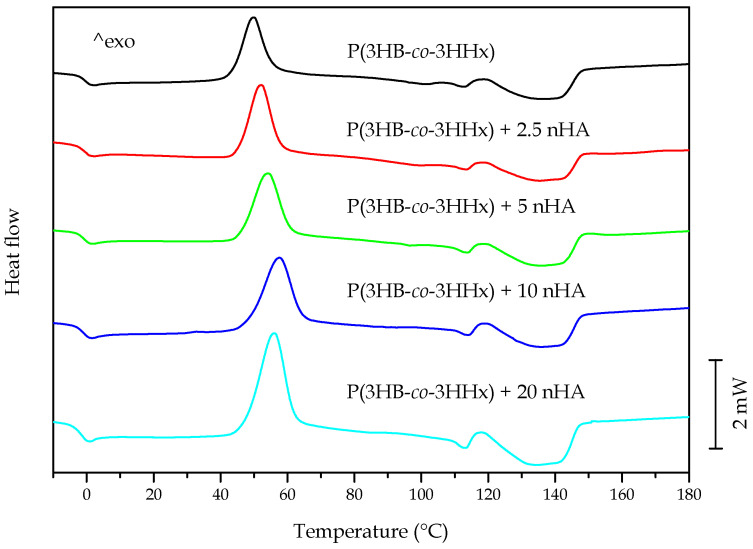
Differential scanning calorimetry (DSC) thermograms taken during second heating of the injection-molded poly(3-hydroxybutyrate-*co*-3-hydroxyhexanoate) [P(3HB-*co*-3HHx)]/hydroxyapatite nanoparticle (nHA) parts.

Figure 3 displays the DSC curves for the neat P(3HB-*co*-3HHx) part and the P(3HB-*co*-3HHx)/nHA composite parts with different nanoparticle contents. Table 5 presents the thermal properties obtained from the second heating scan, after erasing the thermal history of the sample. At approximately 0 °C, one could observe a step change in the base lines, which corresponded to the glass transition temperature (T_g_) of P(3HB-*co*-3HHx). This second-order thermal transition was located at −0.3 °C for the neat biopolymer and it was significantly unaffected by the presence of nHA. The exothermic peaks located between 40 and 70 °C corresponded to the cold crystallization temperature (T_cc_) of P(3HB-*co*-3HHx). In the case of the neat biopolymer part, this peak was located at 49.8 °C. It could be observed that the values of T_cc_ increased with increasing nHA content until 10 wt %, and then slightly decreased at the highest content tested, that is, 20 wt %. These results suggested that low nHA contents impaired the movement of P(3HB-*co*-3HHx) chains and, hence, hindered the crystallization process. A similar thermal behavior during the analysis of the second heating curves was recently observed by Senatov et al. [61], who associated the presence of nHA to a decrease in the molecular chain mobility of the biopolymer that impeded the crystallization process. Finally, the crystalline P(3HB-*co*-3HHx) domains melted in the thermal range from 100 to 150 °C in two peaks. Furthermore, the occurrence of a broad melting region suggested the presence of heterogeneous crystallites with different degrees of perfection, commonly produced in PHAs with relatively high comonomer contents [62]. The thermogram of neat P(3HB-*co*-3HHx) revealed two melting temperatures (T_m1_ and T_m2_) at approximately 113 and 140 °C. Similar thermal properties were reported by Zhou et al. [63] for P(3HB-*co*-3HHx) with 11 mol % content of 3HHx, who also observed a double-melting peak phenomenon in the DSC heating curves of this copolyester. The presence of two melting peaks have been previously ascribed to the melting–recrystallization–melting process of P(3HB-*co*-3HHx) [64]. During this process, imperfect crystals melt at lower temperatures and the amorphous regions order into packed spherulites with thicker lamellar thicknesses that, thereafter, melt at higher temperatures. Alternatively, the melting peaks attained at low temperatures, that is, 110–115 °C, could also relate to the crystalline phase of the 3HHx-rich fractions. Lastly, one could observe that the melting profile of P(3HB-*co*-3HHx) was nearly unaffected by the nHA presence, indicating that the nanoparticles did not significantly influence the crystallization process.

In addition to the characteristic values of T_g_, T_cc_, and T_m_, the enthalpies corresponding to the cold crystallization (ΔH_cc_) and melting (ΔH_m_) enthalpies were collected from the DSC curves. The latter parameter was used to determine the maximum degree of crystallinity, that is, *X_C_max_*, which gives more information about the effect of the additives on the biopolymer, since it does not consider the crystals formed during cold crystallization. It can be seen that P(3HB-*co*-3HHx) showed a maximum degree of crystallinity of 21.4%. One can also observe that crystallinity varied significantly with nHA content. In particular, as nHA was gradually incorporated in higher percentages, the crystallinity increased steadily up to a maximum of nearly 29% at 5 wt % of nHA and then it slightly decreased to values close to 25% for nHA contents of 10 and 20 wt %. This result, in combination with the slightly higher T_cc_ and T_m_ values, suggests that the nanoparticles hindered the formation of crystals at low temperatures, but the crystals formed were slightly more perfect and more mass crystallized. This is in agreement with previous studies that concluded that the introduction of nHA into biopolyesters has an effect on the ordering of their molecular chains by acting as a nucleating agent [61,65].

**Figure 4:**
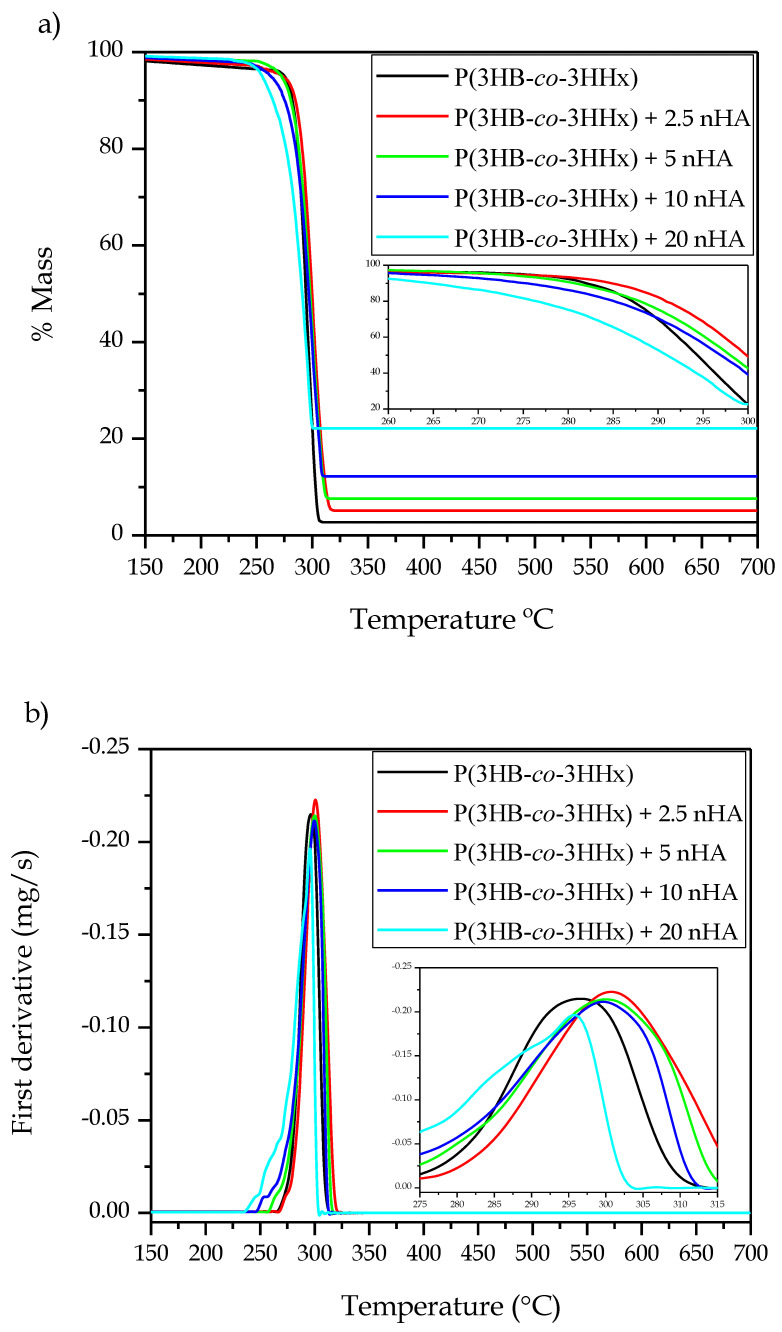
(**a**) Thermogravimetric analysis (TGA) and (**b**) first derivate thermogravimetric (DTG) curves of the injection-molded poly(3-hydroxybutyrate-*co*-3-hydroxyhexanoate) [P(3HB-*co*-3HHx)]/hydroxyapatite nanoparticle (nHA) parts.

Figure 4 presents the thermogravimetric data for all the materials, while Table 6 gathers the main thermal stability parameters obtained from the TGA curves. Thermal degradation of P(3HB-*co*-3HHx) was observed to occur through a one-step process, which is in agreement with the values reported by Li et al. [20], who showed that the thermal stability of the microbial copolyester was as high as 225 °C with almost no mass loss. The temperature at 5% mass loss (T_5%_) showed no significant differences with nHA contents of up to 5 wt %, but a significant decrease was observed for higher loadings. The temperature at which the maximum mass loss rate occurred (T_deg_) increased from 296.7 °C, for the neat P(3HB-*co*-3HHx) part, to 300.9 °C, for the part of P(3HB-*co*-3HHx) filled with 2.5 %wt of nHA. This increase in thermal stability has been previously ascribed to the formation of strong hydrogen interactions and Van der Walls forces between the inorganic nanoparticles and the biopolymer chains during the melt-mixing process [66]. The values of T_deg_ remained nearly constant, showing no significant differences for nHA contents from 2.5 to 10 wt %, but it significantly decreased to 295.6 °C in the part filled with 20 wt % of nHA. The onset of degradation was also reduced for the most filled sample, showing a T_5%_ value of 254.8 °C, which represents a reduction of approximately 18 °C in comparison to the unfilled P(3HB-*co*-3HHx) sample and its nanocomposites at low contents. These results further indicate that the nanoparticles formed aggregates at high contents, which created volumetric gradients of concentration [66]. In this regard, Bikiaris et al. [65] suggested that when high amounts of nanosized filler aggregates are formed, the structure shifts from nanocomposite to microcomposite and, thus, the shielding effect of the nanosized particles is lessened. In addition, Chen et al. [67] reported that high loadings of nHA in PHBV lower the onset degradation temperature since they can catalyze thermal decomposition. In any case, low nHA loadings (<10 wt %) slightly improved the thermal stability of P(3HB-*co*-3HHx) parts and their thermal stability is considered to be high enough for bone tissue engineering and biomedical applications, which can require thermal sterilization methods such as dry heat sterilization (160 °C for 2 h) and steam sterilization (121 °C for 20–60 min) [68]. However, the relatively low T_m_ of P(3HB-*co*-3HHx) would limit the use of these techniques for sterilization and the resultant implantable biomedical devices should be sterilized at low temperatures using ethylene oxide (EO) gas, gamma radiation or ozone. Finally, it can be observed that the residual mass at 700 °C increased gradually with the nHA content due to the high thermal stability of the mineral nanoparticles.

### 3.3. Thermomechanical Characterization of the P(3HB-co-3HHx)/nHA Parts

**Figure 5:**
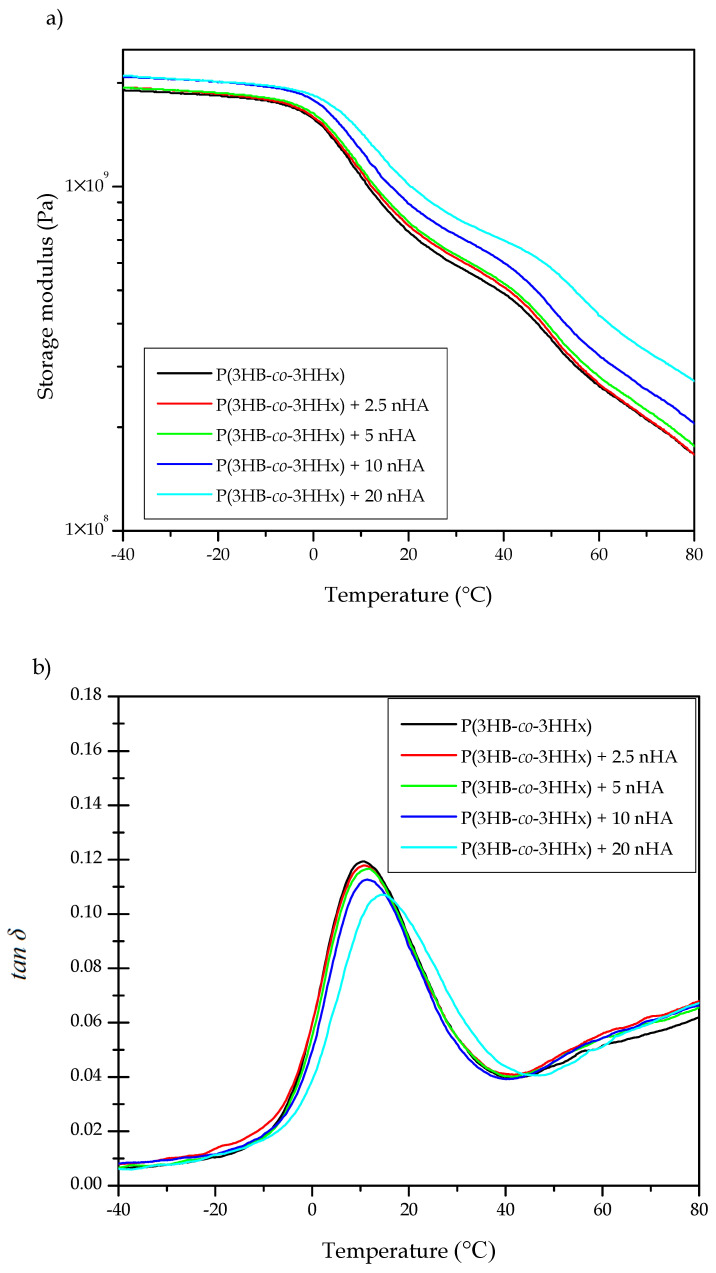
Evolution as a function of temperature of the (**a**) storage modulus and (**b**) dynamic damping factor (*tan δ*) of the injection-molded hydroxybutyrate-*co*-3-hydroxyhexanoate) [P(3HB-*co*-3HHx)]/hydroxyapatite nanoparticles (nHA) parts.

DMTA was carried out on the injection-molded composite parts in order to understand the role played by nHA on the viscoelastic behavior of P(3HB-*co*-3HHx)/nHA. Figure 5 illustrates the DMTA curves of the neat P(3HB-*co*-3HHx) part and the P(3HB-*co*-3HHx)/nHA composite parts with different nanoparticle contents. Figure 5a gathers the evolution of the storage moduli (E’) in the temperature sweep from −40 to 80 °C at a frequency of 1 Hz. The T_g_ values and the corresponding values of E’ at −40, 37, and 70 °C are presented in Table 7, since the first and last temperatures are representative of the stored elastic energy of the amorphous phase of P(3HB-*co*-3HHx) in its glassy and rubber states, respectively, whereas the middle one corresponds to the actual temperature of the human body. It can be observed that all the P(3HB-*co*-3HHx)-based parts presented a similar thermomechanical profile. In particular, the samples showed high E’ values, that is, high stiffness, at temperatures below 0 °C and then E’ sharply decreased. This thermomechanical change was produced because the temperature exceeded the alpha (α)-relaxation of the biopolymer, which is related to its T_g_. One can also observe that the rate of decrease of E’ reduced somewhat when the temperature reached approximately 40 °C due to the occurrence of cold crystallization. The values of E’ at −40, 37, and 70 °C of the neat P(3HB-*co*-3HHx) part were 1909.9, 519.2, and 210.5 MPa, respectively. The E’ value attained at 37 °C was in accordance with the mechanical data presented in Section 3.1, which indicated that only the P(3HB-*co*-3HHx) parts filled with the highest nHA contents, that is, 15 and 20 wt %, showed significantly higher values. However, the results also indicated that the parts crystallized during the ageing process since the thermomechanical changes during and after cold crystallization were relatively low. As expected, the E’ values progressively increased with increasing the nHA content, given the high stiffness of the nanoparticles. It is worth noting that the reinforcing effect was more noticeable at higher temperatures since the amorphous phase of P(3HB-*co*-3HHx) was in the rubber state. Indeed, at higher temperatures, the thermomechanical response of all the P(3HB-*co*-3HHx) composite parts was significantly different, dependent upon the nHA content. For instance, at −40 °C the E’ value increased from 1935.2 MPa for the nanocomposite part containing 2.5 wt % of nHA, to 2100.4 MPa for the part filled with 20 wt % of nHA, whereas these values increased from 212.3 MPa to 333.1 MPa at 70 °C.

The loss tangent or dynamic damping factor (*tan δ*) curves are shown in Figure 5b. Since the position of the *tan δ* peak gives an indication of the biopolymer’s T_g_, these values were also included in Table 7. In the case of the neat P(3HB-*co*-3HHx) part, the *tan δ* peak was located at 10.7 °C, which is similar to that reported by Valentini et al. [69]. It is worth mentioning that, in all cases, the *tan δ* peaks were approximately 10 °C higher than the T_g_ values. Since *tan δ* represents the ratio of the viscous to the elastic response of a viscoelastic material, this indicates that part of the applied load was dissipated by energy dissipation mechanisms such as segmental motions, which are related to T_g_, but part of the energy was also stored and released upon removal of the load at higher temperatures. One can observe that the incorporation of nHA shifted slightly the position of the *tan δ* peaks and also reduced their intensity for the highest nHA loadings, that is, 10 and 20 wt %. Decreasing *tan δ* peaks intensity indicated that the nanocomposite parts showed a more elastic response and, hence, presented more potential to store the applied load rather than dissipating it [70]. This reduction is directly related to the higher E’ values attained due to nanoparticle reinforcement and it confirmed that nHA imposed restrictions on the molecular motion of the P(3HB-*co*-3HHx) chains, resulting in a material with more elastic behavior [71]. It also correlated well with the DSC results shown above, indicating that P(3HB-*co*-3HHx) developed more crystallinity in the nanocomposite parts due to the nucleating effect of nHA and, thus, the less amorphous phase underwent glass transition.

The effect of temperature on the dimensional stability of the P(3HB-*co*-3HHx)/nHA parts was also determined by TMA. The coefficient of linear thermal expansion (CLTE), both below and above T_g_, was obtained from the change in dimensions versus temperature and it is also included in Table 7 along with the T_g_ values. In all cases, lower CLTE values were attained in the parts below T_g_, due to the lower mobility of the P(3HB-*co*-3HHx) chains of the amorphous regions in the glassy state. As anticipated, both below and above T_g_, the CLTE values decreased significantly with increasing nHA content due to the increasing replacement of the soft biopolymer matrix by a ceramic material with a considerably lower CLTE value, that is, 13.6 μm/m·°C [72]. As a result, the CLTE value below T_g_ was reduced from 64.3 μm/m·°C for the neat P(3HB-*co*-3HHx) part, to 56.7 μm/m·°C for the nanocomposite part filled with 20 wt % of nHA. Similarly, above T_g_, it decreased from 177.2 to 159.1 μm/m·°C, respectively. This thermomechanical response was slightly better than that of the PLA/nHA composites, in which the CLTE values below T_g_ decreased from 73 to 71 μm/m·°C after the incorporation of 20 wt % of nHA into PLA μm/m·°C [73]. These results point out that the nanocomposite parts prepared herein show excellent dimensional stability against temperature exposition. However, it is also worth mentioning that, as expected, the CLTE of Ti-based materials was significantly lower, having a mean value of 8.7 μm/m·°C [72].

### 3.4. Morphological Characterization of the P(3HB-co-3HHx)/nHA Parts

**Figure 6:**
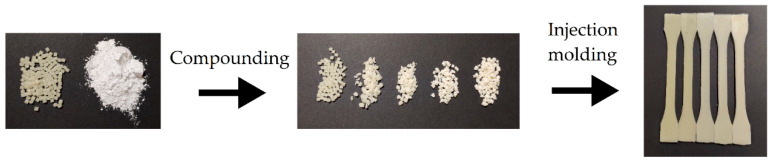
Processing steps carried out to prepare the poly(3-hydroxybutyrate-*co*-3-hydroxyhexanoate) [P(3HB-*co*-3HHx)]/hydroxyapatite nanoparticle (nHA) parts; from left to right: as-received P(3HB-*co*-3HHx) pellets and nHA powder, compounded pellets of the nanocomposite, injection-molded parts.

Figure 6 shows the samples before and after the various processing steps. Combining melt compounding and injection molding represents a cost-competitive melt-processing methodology to produce a large number of parts using nanocomposites. According to this route, the P(3HB-*co*-3HHx) pellets and the nHA powder were pre-mixed and fed together to the co-rotating twin-screw extruder. In this way, pellets of nanocomposites containing different contents of dispersed nHA particles were obtained. They were subsequently injection molded into dumbbell bars. All parts were defect-free and had a bright surface, the nHA content influencing their color; neat P(3HB-*co*-3HHx) parts were yellow pale, typical of microbial PHA, while the presence of nanoparticles induced a whiter color.

**Figure 7:**
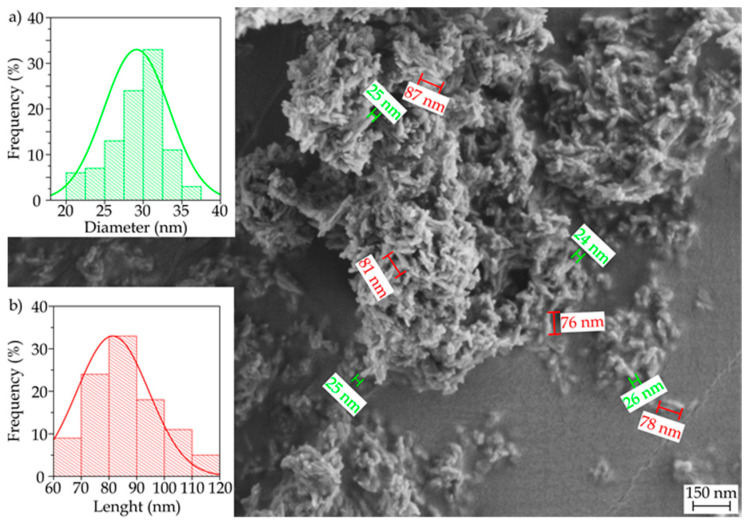
Field-emission scanning electron microscopy (FESEM) images of the hydroxyapatite nanoparticles (nHA) powder. Image was taken at 10,000× with scale marker 150 nm.

Figure 7 shows the FESEM image, taken at 10,000×, of the nHA powder. The nanoparticles show a flake-like morphology based on plates with sizes 60–120 nm and mean cross-sections of approximately 30 nm. This particular morphology of nHA has been reported to occur at pH values below 9, due to the solution environment changes by the OH^−^ ions during synthesis using polyethylene glycol (PEG) as a template [74].

**Figure 8:**
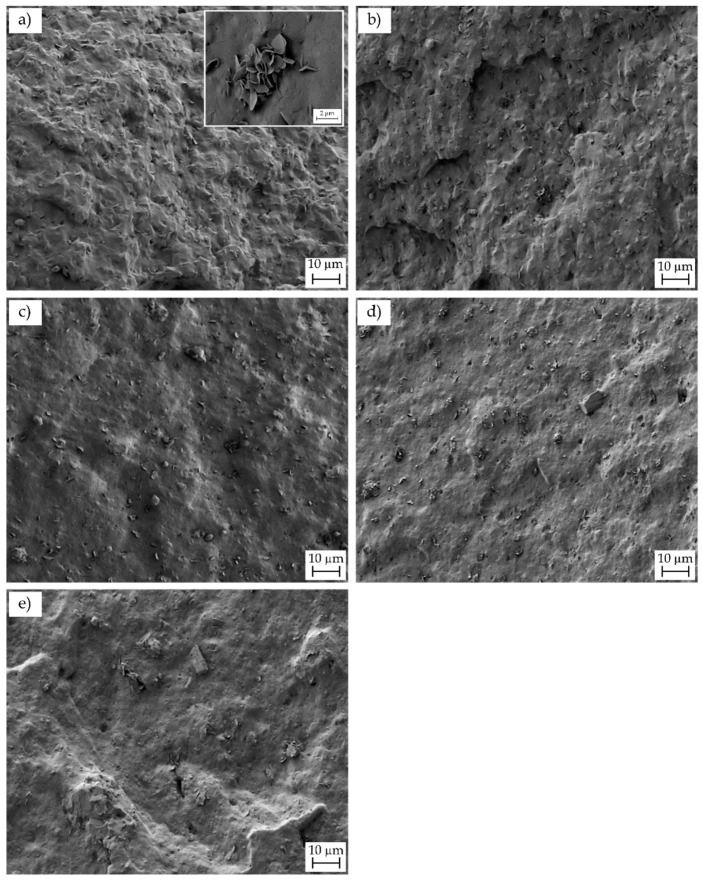
Field-emission scanning electron microscopy (FESEM) images of the fracture surfaces of the injection-molded poly(3-hydroxybutyrate-*co*-3-hydroxyhexanoate) [P(3HB-*co*-3HHx)]/hydroxyapatite nanoparticle (nHA) parts of: (**a**) neat P(3HB-*co*-3HHx); (**b**) P(3HB-*co*-3HHx) + 2.5 nHA; (**c**) P(3HB-*co*-3HHx) + 5 nHA; (**d**) P(3HB-*co*-3HHx) + 10 nHA; (**e**) P(3HB-*co*-3HHx) + 20 nHA. Images were taken at 500× and with scale markers of 10 µm. Inset image showing the detail of the microparticles was taken at 2500× with scale marker of 2 µm.

Micrographs obtained by FESEM of the fracture surfaces of the injection-molded parts of P(3HB-*co*-3HHx) and the various P(3HB-*co*-3HHx)/nHA composites after the Charpy impact tests are gathered in Figure 8. The fracture surface of the neat P(3HB-*co*-3HHx) part, shown in Figure 8a, indicated that the material presented a relatively high toughness, since it yielded a rough surface with the presence of multiple microcracks and some holes. Some microparticles could be seen in the inset FESEM micrograph taken at higher magnification, which could be related to the presence of nucleating agents and/or fillers added by the manufacturer, such as boron nitride (BN). In this regard, Türkez, et al. [75] have recently demonstrated that BN nanoparticles show slight cytotoxicity potential. In particular, contents below 100 mg/L did not lead to lethal response on human primary alveolar epithelial cells (HPAEpiC), suggesting their safe and effective use in both pharmacological and medical applications. Figure 8b–e gather the fracture surfaces of the P(3HB-*co*-3HHx)/nHA composite parts. The morphological characteristics of the fracture surfaces for the nanocomposites filled with low nHA contents, that is, 2.5 and 5 wt %, remained very similar to that of neat P(3HB-*co*-3HHx)/nHA. In all cases, the nanoparticles were relatively well dispersed and distributed within the biopolymer matrix. However, at higher contents, the nanoparticles tended to form some microaggreagates and the resultant fracture surfaces were smoother, indicating that the nanocomposites were more brittle.

**Figure 9:**
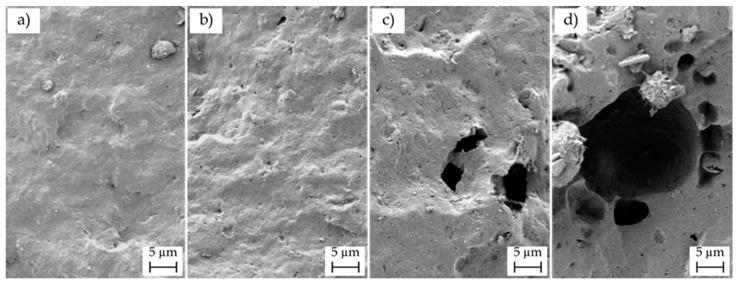
Field-emission scanning electron microscopy (FESEM) images of the fracture surfaces of the injection-molded poly(3-hydroxybutyrate-*co*-3-hydroxyhexanoate) [P(3HB-*co*-3HHx)]/hydroxyapatite nanoparticles (nHA) parts after selective attack with 6M hydrochloric acid (HCl) for 12 h: (**a**) P(3HB-*co*-3HHx) + 2.5 nHA; (**b**) P(3HB-*co*-3HHx) + 5 nHA; (**c**) P(3HB-*co*-3HHx) + 10 nHA; (**d**) P(3HB-*co*-3HHx) + 20 nHA. Images were taken at 1000× with scale marker of 5 µm.

Due to the low nHA particle size and the presence of BN and/or additives in the P(3HB-*co*-3HHx) matrix, selective separation was carried out on the fracture surfaces of the nanocomposite parts, in order to better evaluate the dispersion of the nanoparticles. Figure 9 presents the FESEM images of the fracture surfaces subjected to treatment with 6 M HCl for 12 h. The voids and holes formed in the surfaces were related to removed/dissolved nHA and the overall void size and distribution gave an indication of the original particle dispersion. The micrographs revealed that some microholes were produced after the selective attack on the P(3HB-*co*-3HHx) parts filled with 10 and 20 wt % of nHA, which should correspond to nHA aggregates, whereas the nanocomposites containing low nanoparticle loadings showed nano-sized holes well distributed along the biopolymer matrix, which suggested an efficient dispersion. Agglomeration was particularly noticeable for the nanocomposite part containing 20 wt % of nHA, thus indicating that the presence of aggregates could induce particle debonding during fracture, as a result of the dissimilar mechanical strength and rigidity of the ceramic nanoparticles and biopolymer matrix. Therefore, the present results correlate well with the mechanical and thermal properties described above, in which nHA loadings of up to 10 wt % increased the mechanical and thermal performance of the P(3HB-*co*-3HHx) parts, whereas the highest nHA content impaired the overall properties due to nanoparticle aggregation.

## 4. Conclusions

One of the most exciting areas of new material development in the biomedical device community is resorbable polymers. As bone scaffolds, biodegradable and biocompatible polymers will maintain their strength until the liquid in contact begins the dissolution process, eventually leading to their complete elimination from the body, thus avoiding a second surgery for their removal. The herein-prepared injection-molded composite parts of P(3HB-*co*-3HHx)/nHA showed a better matching of mechanical and thermomechanical performance than metal alloys to replace natural bone. While natural bone has a modulus ranging from about 8–25 GPa, the herein-prepared injection-molded parts showed E_t_ values from approximately 1 up to 1.7 GPa and ε_b_ values ranging from 6.5 to 19.4%. The incorporation of up to 10 wt % of nHA also improved slightly the thermal stability of the P(3HB-*co*-3HHx) parts and their thermal stability was considered to be high enough for bone tissue engineering, taking into account that nonthermal sterilization methods would be required. These balanced properties in terms of strength and ductility offer the biomedical industry a material that can accomplish different applications in bone reconstruction, for which high-stress materials are not needed, such as bone screws and small orthopedic plates or rods. Future works will explore the potential use of the P(3HB-*co*-3HHx)/nHA composites as drug delivery systems.

## Figures and Tables

**Table 1:** Code and composition of the samples prepared according to the weight content (wt %) of poly(3-hydroxybutyrate-*co*-3-hydroxyhexanoate) [P(3HB-*co*-3HHx)] and hydroxyapatite nanoparticles (nHA).

Sample	P(3HB-*co*-3HHx) (wt %)	nHA (wt %)
P(3HB-*co*-3HHx)	100	0
P(3HB-*co*-3HHx) + 2.5 nHA	97.5	2.5
P(3HB-*co*-3HHx) + 5 nHA	95	5
P(3HB-*co*-3HHx) + 10 nHA	90	10
P(3HB-*co*-3HHx) + 20 nHA	80	20

**Table 2:** Number of tested samples (*n*) for each injection-molded poly(3-hydroxybutyrate-*co*-3-hydroxyhexanoate) [P(3HB-*co*-3HHx)]/hydroxyapatite nanoparticles (nHA) parts and the type of statistical test performed for each testing method with level of significance (*p*).

Testing Method	*n*	Normality Test	*p*	Significance Test	*p*
Tensile	6	Shapiro–Wilk	0.05	Tukey	0.05
Flexural	6	Shapiro–Wilk	0.05	Tukey	0.05
Hardness	7	Shapiro–Wilk	0.05	Tukey	0.05
Impact strength	8	Shapiro–Wilk	0.05	Tukey	0.05
DSC	3	-	-	Kruskal-Wallis	0.05
TGA	3	-	-	Kruskal-Wallis	0.05
DMTA	3	-	-	Kruskal-Wallis	0.05
TMA	3	-	-	Kruskal-Wallis	0.05

DSC = differential scanning calorimetry; TGA = thermogravimetric analysis; DMTA = dynamic thermomechanical analysis; TMA = thermomechanical analysis.

**Table 3:** Mechanical properties of the injection-molded parts of poly(3-hydroxybutyrate-*co*-3-hydroxyhexanoate) [P(3HB-*co*-3HHx)]/hydroxyapatite nanoparticles (nHA) in terms of maximum tensile stress (σ_max_), tensile modulus (E_t_), elongation at break (ε_b_), maximum flexural stress (σ_f_), flexural modulus (E_f_), Shore D hardness, and impact strength.

Part	σ_max_ (MPa)	E_t_ (MPa)	ε_b_ (%)	σ_f_ (MPa)	E_f_ (MPa)	Shore D Hardness	Impact Strength (kJ/m^2^)
P(3HB-*co*-3HHx)	17.7 ± 1.1	1022.3 ± 59.2	19.4 ± 0.8	24.1 ± 1.9	735.3 ± 43.5	64.2 ± 0.8	5.1 ± 0.3
P(3HB-*co*-3HHx) + 2.5 nHA	16.1 ± 0.5 *	1097.0 ± 52.3 *	12.9 ± 0.3 *	25.6 ± 1.0 *	744.1 ± 26.3	64.0 ± 0.9	3.5 ± 0.2 *
P(3HB-*co*-3HHx) + 5 nHA	15.8 ± 0.6	1113.1 ± 28.3	12.5 ± 0.8	26.4 ± 1.8	813.2 ± 24.1 *	65.2 ± 0.8 *	2.6 ± 0.2 *
P(3HB-*co*-3HHx) + 10 nHA	15.5 ± 0.5	1398.2 ± 68.3 *	10.4 ± 0.6 *	26.7 ± 0.5	919.8 ± 38.6 *	65.8 ± 1.1	2.2 ± 0.1 *
P(3HB-*co*-3HHx) + 20 nHA	14.2 ± 0.2 *	1681.4 ± 56.3 *	6.5 ± 0.7 *	26.9 ± 1.9	1182.5 ± 45.2 *	69.4 ± 0.5 *	1.7 ± 0.2 *

* Indicates a significant difference compared with the previous sample (*p* < 0.05). Level of significance (*p*) values are included in Table S1.

**Table 4:** Spearman’s test correlation coefficient (*r_s_*) and level of significance (*p*) for each mechanical property.

Mechanical Properties	*r_s_*	*p*
σ_max_ (MPa)	−0.917	0.028
E_t_ (MPa)	+0.988	0.002
ε_b_ (%)	−0.903	0.035
σ_f_ (MPa)	+0.782	0.118
E_f_ (MPa)	+0.993	0.001
Shore D hardness	+0.977	0.004
Impact strength (kJ/m^2^)	−0.839	0.032

**Table 5:** Thermal properties of the injection-molded poly(3-hydroxybutyrate-*co*-3-hydroxyhexanoate) [P(3HB-*co*-3HHx)]/hydroxyapatite nanoparticle (nHA) parts in terms of glass transition temperature (T_g_), cold crystallization temperature (T_cc_), melting temperatures (T_m1_ and T_m2_), cold crystallization enthalpy (∆H_cc_), melting enthalpy (∆H_m_), and maximum degree of crystallinity (χ_c max_).

Part	T_g_ (°C)	T_cc_ (°C)	T_m1_ (°C)	T_m2_ (°C)	ΔH_cc_ (J/g)	ΔH_m_ (J/g)	X_c max_ (%)
P(3HB-*co*-3HHx)	−0.3 ± 0.1	49.8 ± 0.5	112.9 ± 0.5	139.7 ± 0.3	20.7 ± 0.5	31.2 ± 0.4	21.4 ± 1.8
P(3HB-*co*-3HHx) + 2.5 nHA	−0.2 ± 0.1	52.1 ± 0.4	113.6 ± 0.3	140.9 ± 0.4	29.8 ± 0.4 *	35.0 ± 0.5 *	24.3 ± 2.4 *
P(3HB-*co*-3HHx) + 5 nHA	−0.4 ± 0.2	54.1 ± 0.2	113.8 ± 0.4	139.4 ± 0.2	31.8 ± 0.6 *	40.1 ± 0.3 *	28.9 ± 2.2 *
P(3HB-*co*-3HHx) + 10 nHA	−0.4 ± 0.2	57.4 ± 0.3 *	114.3 ± 0.2	139.0 ± 0.3	29.0 ± 0.5 *	32.5 ± 0.4 *	24.7 ± 1.5 *
P(3HB-*co*-3HHx) + 20 nHA	−0.3 ± 0.1	55.9 ± 0.4 *	113.4 ± 0.5	139.1 ± 0.4	25.9 ± 0.4 *	29.0 ± 0.1 *	24.8 ± 1.4

* Indicates a significant difference compared with the previous sample (*p* < 0.05). Level of significance (*p*) values are included in Table S2.

**Table 6:** Main thermal degradation parameters of the injection-molded poly(3-hydroxybutyrate-*co*-3-hydroxyhexanoate) [P(3HB-*co*-3HHx)]/hydroxyapatite nanoparticle (nHA) parts in terms of onset temperature of degradation (T_5%_), degradation temperature (T_deg_), and residual mass at 700 °C.

Part	T_5%_ (°C)	T_deg_ (°C)	Residual Mass (%)
P(3HB-*co*-3HHx)	272.5 ± 2.3	296.7 ± 1.4	2.6 ± 0.3
P(3HB-*co*-3HHx) + 2.5 nHA	272.2 ± 1.7	300.9 ± 2.2	5.1 ± 0.5 *
P(3HB-*co*-3HHx) + 5 nHA	272.4 ± 1.3	299.9 ± 1.7	7.6 ± 0.4 *
P(3HB-*co*-3HHx) + 10 nHA	262.3 ± 1.8 *	299.6 ± 1.8	12.2 ± 0.7 *
P(3HB-*co*-3HHx) + 20 nHA	254.8 ± 1.3 *	295.6 ± 1.6 *	22.1 ± 0.8 *

* Indicates a significant difference compared with the previous sample (*p* < 0.05). Level of significance (*p*) values are included in Table S3.

**Table 7:** Thermomechanical properties of the injection-molded poly(3-hydroxybutyrate-*co*-3-hydroxyhexanoate) [P(3HB-*co*-3HHx)]/hydroxyapatite nanoparticles (nHA) parts in terms of dynamic damping factor (*tan δ*) peak, glass transition temperature (T_g_), storage modulus (E’) measured at −40, 37, and 70 °C, and coefficient of linear thermal expansion (CLTE) below and above *T_g_*.

Part	DMTA				TMA		
	*tan δ* Peak (°C)	E’ at −40 °C (MPa)	E’ at 37 °C (MPa)	E’ at 70 °C (MPa)	*T_g_* (°C)	CLTE (µ/m·°C)	
						Below T_g_	Above T_g_
P(3HB-*co*-3HHx)	10.7 ± 0.4	1909.9 ± 50.2	519.2 ± 14.2	210.5 ± 2.5	−0.6 ± 0.2	64.3 ± 1.1	177.2 ± 4.6
P(3HB-*co*-3HHx) + 2.5 nHA	10.9 ± 0.2	1935.2 ± 41.7 *	544.1 ± 26.3 *	212.3 ± 3.1	−0.3 ± 0.2	61.3 ± 0.4 *	176.1 ± 7.2 *
P(3HB-*co*-3HHx) + 5 nHA	11.2 ± 0.3	1940.1 ± 82.5 *	557.8 ± 17.1 *	222.5 ± 4.6 *	−0.1 ± 0.1	59.3 ± 0.5	175.0 ± 0.8
P(3HB-*co*-3HHx) + 10 nHA	11.4 ± 0.5	2090.3 ± 74.6 *	639.0 ± 18.1 *	256.8 ± 5.1 *	−0.4 ± 0.2	58.2 ± 0.4	170.2 ± 3.8 *
P(3HB-*co*-3HHx) + 20 nHA	14.5 ± 0.4 *	2100.4 ± 65.1 *	728.8 ± 26.6 *	333.1 ± 3.4 *	−0.3 ± 0.2	56.7 ± 0.7	159.1 ± 5.7 *

* Indicates a significant difference compared with the previous sample (*p* < 0.05). Level of significance (*p*) values are included in Table S4.

## Supplementary Materials

The following tables are available online at https://www.mdpi.com/2073-4360/12/6/1389/s1, Table S1: Level of significance (*p*) values for Table 1; Table S2: Level of significance (*p*) values for Table 2; Table S3: Level of significance (*p*) values for Table 3; Table S4: Level of significance (*p*) values for Table 4.
