# Supplementary material for: Assessment of the Mechanical and Thermal Properties of Injection-Molded Poly(3-hydroxybutyrate-co-3-hydroxyhexanoate)/Hydroxyapatite Nanoparticles Parts for Use in Bone Tissue Engineering
Source: Polymers (Basel). 2020 Jun 21;12(6):1389. doi: 10.3390/polym12061389 (PMC7362193; doi:10.3390/polym12061389)
Supplement: Supplementary file 1 [file polymers-12-01389-s001.pdf]

**Level of significance (*p*) values for Table 1.** Mechanical properties of the injection-molded parts of poly(3-hydroxybutyrate-*co*-3-hydroxyhexanoate) [P(3HB-*co*-3HHx)]/hydroxyapatite nanoparticles (nHA) in terms of maximum tensile stress ($\sigma_{max}$), tensile modulus ($E_t$), elongation at break ($\varepsilon_b$), maximum flexural stress ($\sigma_f$), flexural modulus ($E_f$), Shore D hardness, and impact strength.

| Part | $\sigma_{max}$ (MPa) | $E_t$ (MPa) | $\varepsilon_b$ (%) | $\sigma_f$ (MPa) | $E_f$ (MPa) | Shore D hardness | Impact Strength (kJ/m²) |
|---|---|---|---|---|---|---|---|
| P(3HB-*co*-3HHx) | - | - | - | - | - | - | - |
| P(3HB-*co*-3HHx) + 2.5 nHA | 0.056 | 0.080 | 0.309 | 0.052 | 0.032 | 0.027 | 0.285 |
| P(3HB-*co*-3HHx) + 5 nHA | 0.012 | 0.032 | 0.031 | 0.039 | 0.090 | 0.043 | 0.218 |
| P(3HB-*co*-3HHx) + 10 nHA | 0.016 | 0.158 | 0.163 | 0.014 | 0.122 | 0.037 | 0.115 |
| P(3HB-*co*-3HHx) + 20 nHA | 0.066 | 0.204 | 0.319 | 0.011 | 0.284 | 0.067 | 0.248 |

**Level of significance (*p*) values for Table 2.** Thermal properties of the injection-molded poly(3-hydroxybutyrate-*co*-3-hydroxyhexanoate) [P(3HB-*co*-3HHx)]/hydroxyapatite nanoparticles (nHA) parts in terms of glass transition temperature ($T_g$), cold crystallization temperature ($T_{cc}$), melting temperatures ($T_{m1}$ and $T_{m2}$), cold crystallization enthalpy ($\Delta H_{cc}$), melting enthalpy ($\Delta H_m$), and maximum degree of crystallinity ($\chi_{c\,max}$).

| Part | $T_g$ (°C) | $T_{cc}$ (°C) | $T_{m1}$ (°C) | $T_{m2}$ (°C) | $\Delta H_{cc}$ (J/g) | $\Delta H_m$ (J/g) | $X_{c\,max}$ (%) |
|---|---|---|---|---|---|---|---|
| P(3HB-*co*-3HHx) | - | - | - | - | - | - | - |
| P(3HB-*co*-3HHx) + 2.5 nHA | 0.002 | 0.041 | 0.009 | 0.011 | 0.443 | 0.095 | 0.090 |
| P(3HB-*co*-3HHx) + 5 nHA | 0.003 | 0.030 | 0.004 | 0.013 | 0.051 | 0.128 | 0.186 |
| P(3HB-*co*-3HHx) + 10 nHA | 0.001 | 0.059 | 0.006 | 0.005 | 0.052 | 0.142 | 0.132 |
| P(3HB-*co*-3HHx) + 20 nHA | 0.002 | 0.051 | 0.010 | 0.003 | 0.082 | 0.099 | 0.010 |

**Level of significance (*p*) values for Table 3.** Main thermal degradation parameters of the injection-molded poly(3-hydroxybutyrate-*co*-3-hydroxyhexanoate) [P(3HB-*co*-3HHx)]/hydroxyapatite nanoparticles (nHA) parts in terms of onset temperature of degradation ($T_{5\%}$), degradation temperature ($T_{deg}$), and residual mass at 700 °C.

| Part | $T_{5\%}$ (°C) | $T_{deg}$ (°C) | Residual mass (%) |
|---|---|---|---|
| P(3HB-*co*-3HHx) | - | - | - |
| P(3HB-*co*-3HHx) + 2.5 nHA | 0.021 | 0.041 | 0.867 |
| P(3HB-*co*-3HHx) + 5 nHA | 0.025 | 0.028 | 0.404 |
| P(3HB-*co*-3HHx) + 10 nHA | 0.058 | 0.025 | 0.555 |
| P(3HB-*co*-3HHx) + 20 nHA | 0.051 | 0.059 | 0.793 |

**Level of significance ($p$) values for Table 4.** Thermomechanical properties of the injection-molded poly(3-hydroxybutyrate-*co*-3-hydroxyhexanoate) [P(3HB-*co*-3HHx)]/hydroxyapatite nanoparticles (nHA) parts in terms of dynamic damping factor (*tan δ*) peak, glass transition temperature ($T_g$), storage modulus (E′) measured at -40 °C, 37 °C, and 70 °C, and coefficient of linear thermal expansion (CLTE) below and above $T_g$.

| Part | DMTA | | | | TMA | | |
|---|---|---|---|---|---|---|---|
| | *tan δ* peak (°C) | E′ at -40 °C (MPa) | E′ at 37 °C (MPa) | E′ at 70 °C (MPa) | $T_g$ (°C) | CLTE (μ/m·°C) | |
| | | | | | | Below $T_g$ | Above $T_g$ |
| P(3HB-*co*-3HHx) | - | - | - | - | - | - | - |
| P(3HB-*co*-3HHx) + 2.5 nHA | 0.021 | 0.280 | 0.153 | 0.027 | 0.002 | 0.051 | 0.078 |
| P(3HB-*co*-3HHx) + 5 nHA | 0.031 | 0.397 | 0.130 | 0.068 | 0.001 | 0.038 | 0.014 |
| P(3HB-*co*-3HHx) + 10 nHA | 0.023 | 0.581 | 0.243 | 0.141 | 0.002 | 0.023 | 0.065 |
| P(3HB-*co*-3HHx) + 20 nHA | 0.226 | 0.400 | 0.260 | 0.295 | 0.001 | 0.033 | 0.122 |
